# The Severity of Intracranial Hemorrhages Measured by Free Hemoglobin in the Brain Depends on the Anticoagulant Class: Experimental Data

**DOI:** 10.1155/2017/6516401

**Published:** 2017-07-20

**Authors:** Kyle M. Ware, Douglas L. Feinstein, Israel Rubinstein, Prudhvi Battula, Jose Otero, Lee Hebert, Tzu-Fei Wang, Alexandra Ivanova, Shweta Chaudhary, Jessica Hemminger, Sergey V. Brodsky

**Affiliations:** ^1^Department of Pathology, The Ohio State University, Columbus, OH, USA; ^2^Department of Anesthesiology, University of Illinois at Chicago, Chicago, IL, USA; ^3^Jesse Brown VA Medical Center, Chicago, IL, USA; ^4^Department of Medicine, University of Illinois at Chicago, Chicago, IL, USA; ^5^Department of Medicine, The Ohio State University, Columbus, OH, USA; ^6^OhioHealth Rehabilitation Hospital, Columbus, OH, USA

## Abstract

*Background and Purpose. *Anticoagulant therapy is broadly used to prevent thromboembolic events. Intracranial hemorrhages are serious complications of anticoagulation, especially with warfarin. Direct oral anticoagulants reduce but do not eliminate the risk of intracranial hemorrhages. The aim of this study is to determine the degree of intracranial hemorrhage after application of anticoagulants without additional triggers.* Methods. *Rats were treated with different anticoagulant classes (vitamin K antagonists, heparin, direct thrombin inhibitor, and factor Xa inhibitor). Brain hemorrhages were assessed by the free hemoglobin concentration in the brain parenchyma.* Results. *Vitamin K antagonists (warfarin and brodifacoum) significantly increased free hemoglobin in the brain. Among direct oral anticoagulants, thrombin inhibitor dabigatran also significantly increased free hemoglobin in the brain, whereas treatment with factor Xa inhibitor rivaroxaban did not have significant effect on the free hemoglobin concentration.* Conclusions*. Our data indicates that the severity of brain hemorrhages depends on the anticoagulant class and it is more pronounced with vitamin K antagonists.

## 1. Introduction

Anticoagulant therapy is the standard of care in patients at high risk for thromboembolic events, most notably with atrial fibrillation or venous thromboembolism. Warfarin is still the most commonly prescribed anticoagulant, with rapid market uptake of direct oral anticoagulants (DOAC) in recent years. Warfarin provides reliable protection against thromboembolic events. However, this benefit comes at a cost, which is a hemorrhage resulting from warfarin-related coagulopathy. Perhaps the most feared hemorrhagic event related to warfarin is intracranial hemorrhage. The incidence of warfarin-associated intracranial hemorrhage is 1–5% and it accounts for 10–12% of all intracranial hemorrhages. The mortality rate in these patients is as high as 50% [1]. DOAC, such as direct thrombin inhibitors (dabigatran) and factor Xa inhibitors (rivaroxaban and apixaban), reduce but do not completely prevent the risk of intracranial hemorrhages [2].

The aim of this study was to investigate whether intracranial hemorrhages are dependent on the class of anticoagulant in experimental animals.

## 2. Material and Methods

All experiments were conducted in accordance with the NIH Guide for the Care and Use of Laboratory Animals and approved by the Institutional Animal Care and Use Committee. Adult male Sprague Dawley rats (180–220 g, Harlan Laboratories, Indianapolis, IN) were provided food and water ad libitum. Animals were randomized to the treatment groups.

Several anticoagulant classes were used: (1) vitamin K antagonists: brodifacoum (BDF) and warfarin (both from Sigma-Aldrich, St. Louis, MO); (2) direct thrombin inhibitor dabigatran (Pradaxa, Boehringer Ingelheim Pharmaceuticals, Inc., Ridgefield, CT); (3) factor Xa antagonist rivaroxaban (Xarelto, Janssen Pharmaceuticals, Inc, Beerse, Belgium); (4) indirect thrombin and factor Xa inhibitor heparin (Fresenius Kabi USA, Schaumburg, IL). The following routes and doses were used for the medications: BDF: oral gavage, single administration 0.4 mg/kg (median lethal dose, LD50) [3]; warfarin per os in drinking water 2 mg/kg/day for 5 days [4]; dabigatran: oral gavage, single administration 150 mg/kg (LD50) [5]; rivaroxaban: oral gavage, single administration 20 mg/kg (LD50) [6]; heparin: subcutaneous single injection 100 KU/kg (LD50, MSD). These doses are based on different pharmacokinetics and pharmacodynamics of these drugs in rats as compared to humans.

Treatment with anticoagulants was performed for 5 days for most of the drugs (except for warfarin that was administered for 7 days). Mortality rate was recorded; survived animals were sacrificed at day 5 (day 7 for warfarin in order to achieve desired increase in prothrombin time (PT)).

PT was measured daily in the blood obtained from the tail as we described earlier [3, 4, 7]. All rats (died from excessive anticoagulation and survived rats) underwent autopsy; a craniotomy was performed, and the brain was extracted and cut at the middle sagittal plane. The left half of the brain was divided into 3 parts: anterior brain (including the frontal lobes), posterior brain (including the parietal, temporal, and occipital lobes), and the cerebellum. These 3 portions of the brain were frozen at −80°C for further evaluation of free hemoglobin.

Intracranial hemorrhages were assessed by histologic examination of the brains and the free hemoglobin concentration in the brain parenchyma. Free hemoglobin was measured using Drabkin's reagent based on a modified manufacturer protocol. The total hemoglobin concentration was calculated per gm of brain tissue from the calibration curve.

### 2.1. Statistics

Statistical analysis was performed using a Prism software (GraphPad Software, Inc., La Jolla, CA). The differences between groups were analyzed by analysis of variance (ANOVA) test followed by postanalysis Tukey's Multiple Comparison Test. Differences with *p* < 0.05 were considered statistically significant.

## 3. Results

Treatments with BDF and heparin resulted in at least 50% mortality (day 2 for BDF and day 1 for heparin). Treatments with other anticoagulants were not fatal. Treatment with vitamin K antagonists resulted in a rapid increase in PT and sINR above 4 [3].

**Figure 1:**
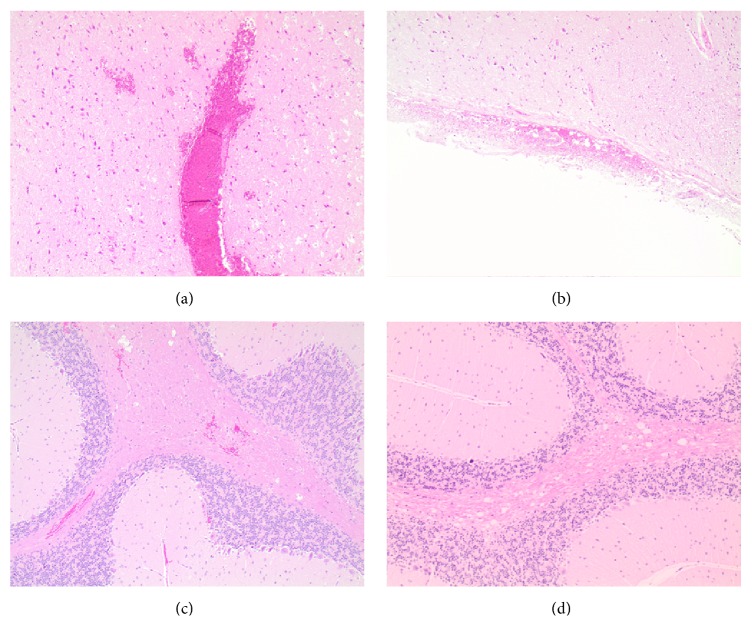
*Histologic findings in the brain in rats treated with brodifacoum*. (a) Vascular congestion, perivascular and interstitial hemorrhage, anterior brain, ×100. (b) Subarachnoidal hemorrhage, posterior brain, ×100. (c) Interstitial hemorrhage, cerebellum, ×100. (d) Control rat, cerebellum, ×100.

Intracranial hemorrhages were not appreciated grossly at autopsy in either of the treatment groups. However, microscopic examination showed microscopic interstitial hemorrhages in animals treated with BDF, but not other anticoagulants (Figures 1(a)–1(c)).

**Figure 2:**
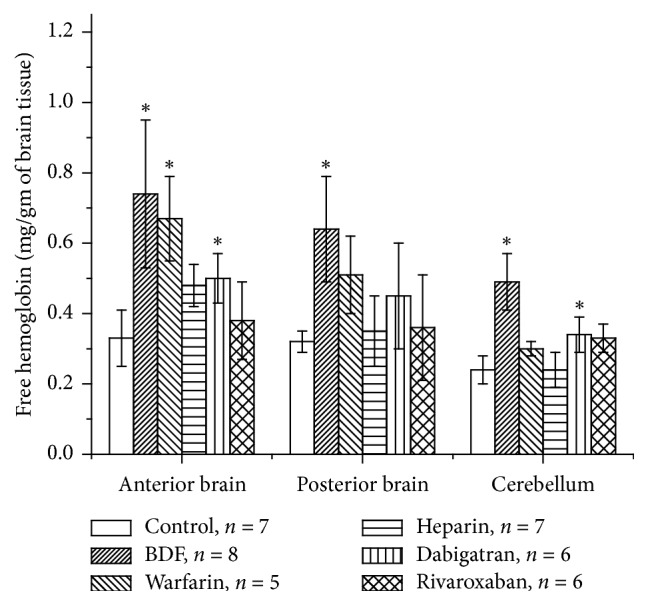
*Free hemoglobin concentration in the brain tissue in rats treated with different anticoagulants*. Hemoglobin concentration was measured as mg of free hemoglobin per gram of the brain tissue. ^*∗*^*p* < 0.05 compared to control.

Results of free hemoglobin concentration measurements are shown in Figure 2. Treatments with BDF lead to a significant increase in free hemoglobin in all three compartments of the brain. Another vitamin K antagonist, warfarin, resulted in a significant increase in the free hemoglobin concentration in the anterior brain only. Free hemoglobin concentration in the posterior brain in animals treated with warfarin increased, but it did not reach statistically significant differences (Figure 2). Treatment with direct thrombin inhibitor dabigatran resulted in an increase in the free hemoglobin concentrations in all 3 brain compartments, but it was statistically significant only in the anterior brain and the cerebellum. Neither heparin nor factor Xa inhibitor rivaroxaban significantly increased free hemoglobin in the brain (Figure 2).

## 4. Discussion

Our work is the first to describe differences in the spontaneous brain hemorrhages after treatments with different anticoagulant classes. Previous works were focused mostly on effects of different anticoagulants in experimental (induced) intracranial hemorrhages.

Anticoagulation is a life-saving therapy and it is widely used in clinical practice. Both vitamin K antagonists and DOAC are associated with intracranial hemorrhages with high mortality rate [2]. Data regarding mechanisms of these intracranial hemorrhages are controversial. It is also not clear whether these hemorrhages are associated with excessive anticoagulation or they are the result of anticoagulation itself. It has been shown at hospital admissions that most patients with warfarin-associated intracranial hemorrhages had therapeutic ranges of INR. However, higher INR has been associated with an increased mortality rate in patients who developed intracranial hemorrhage [8]. Our findings support these clinical observations. The most significant increase in the free hemoglobin concentration in the brain was seen in animals treated with vitamin K antagonists. When anticoagulation associated with vitamin K deficiency was severe (as in case with BDF), the increase in free hemoglobin in the brain was more significant and seen in all brain compartments. On the other hand, when anticoagulation with vitamin K antagonist was similar to those seen in the clinical practice (INR increase 4-5-fold), then the significant increase in the free hemoglobin was noted only in the anterior brain, whereas in the posterior brain or the cerebellum increase in free hemoglobin was not significant (Figure 2). Indeed, it has been demonstrated that warfarin-associated intracranial hemorrhages in patients are predominantly intraparenchymal [1]. Interestingly, direct thrombin inhibitor dabigatran leads to significant increases in free hemoglobin not only in the anterior brain, but in the cerebellum as well. Another DOAC, factor Xa inhibitor, did not increase the concentration of free hemoglobin in either brain compartment, but there was a trend in increased free hemoglobin in the cerebellum (Figure 2).

Our data is supported by previous observations. It has been shown that warfarin significantly increases the blood brain barrier (BBB) permeability [9] and secondary hemorrhage after thrombolysis [10] or collagenase-induced intracranial hemorrhage [11]. There is data that rivaroxaban does not increase hemorrhage after thrombolysis in experimental ischemic stroke in mice [9]; however, other studies indicate that rivaroxaban substantially increases the hematoma volume in intracranial hemorrhage induced by collagenase in mice [12]. The same group reported similar results after treatment of experimental animals with dabigatran [13]. On the other hand, dabigatran did not significantly increase hemorrhagic transformation after transient focal cerebral ischemia in mice [14] or risk of secondary hemorrhages after thrombolysis in various rodent models of ischemia and reperfusion [10] or collagenase-induced intracranial hemorrhage [11]. We and others reported that warfarin and dabigatran both induce glomerular hemorrhage in the kidney [4, 15].

Limitations of our studies include a single supratherapeutic dose of anticoagulants and difficulties to compare the degree of anticoagulation. We used LD50 doses that have been previously described for rats; these doses are significantly higher than ones used in the clinical setting for humans; therefore, more detailed studies that will include dose-response effects of these and other anticoagulants are warranted.

In summary, our experimental data confirm recent clinical trials outcomes that DOAC reduce but do not completely prevent intracranial hemorrhages associated with anticoagulant treatment. Among DOAC, factor Xa inhibitor rivaroxaban results in lower free hemoglobin concentration in the brain when compared to direct thrombin inhibitor dabigatran.
